# Physical Activity Elements and Adverse Outcomes in Patients with Chronic Kidney Disease in Guangdong (PEAKING) project: protocol for a prospective cohort study

**DOI:** 10.1136/bmjopen-2024-086509

**Published:** 2024-10-22

**Authors:** Changyuan Yang, Ruolan Duan, Zhenhua Yang, Jiamei Qiu, Minhui Pi, Xitao Ling, Cuixia Xiao, Jiahao Zeng, Jiawei He, Jiasheng Huang, La Zhang, Xindong Qin, Fang Tang, Lizhe Fu, Haijing Hou, Xusheng Liu, Bengt Lindholm, Fuhua Lu, Yifan Wu, Guobin Su, PEAKING study investigators, Shujuan Chen

**Affiliations:** 1 State Key Laboratory of Traditional Chinese Medicine Syndrome, Chinese Medicine Guangdong Laboratory, Guangdong Provincial Key Laboratory of Chinese Medicine for Prevention and Treatment of Refractory Chronic Diseases, Big Data Research Center of Chinese Medicine, Department of Nephrology, the Second Clinical College of Guangzhou University of Chinese Medicine, Guangdong Provincial Hospital of Chinese Medicine, Guangzhou, China; 2 Department of Nephrology, University Medical Centre Groningen, University of Groningen, Groningen, The Netherlands; 3 Department of Nephrology, Peking University First Hospital, Peking University, Beijing, China; 4 Department of Nephrology, Shenzhen Hospital of Guangzhou University of Chinese Medicine, Shenzhen, China; 5 Chronic Disease Management Center, The Second Affiliated Hospital of Guangzhou University of Chinese Medicine, Guangzhou, China; 6 Division of Renal Medicine and Baxter Novum, Department of Clinical Science, Intervention and Technology, Karolinska Institutet, Stockholm, Sweden; 7 Department of Medical Epidemiology and Biostatistics, Karolinska Institutet, Stockholm, Sweden; 8 Nuffield Department of Population Health, University of Oxford, Oxford, UK

**Keywords:** Mortality, Nephrology, Chronic renal failure, Physical Examination

## Abstract

**Abstract:**

**Introduction:**

Physical inactivity is prevalent and associated with adverse outcomes among patients with chronic kidney disease (CKD). Most previous studies have relied on subjective questionnaires to assess levels of physical activity (PA) and mainly focused on patients undergoing dialysis. Therefore, the Physical Activity Elements and Adverse Outcomes in Patients with Chronic Kidney Disease in Guangdong study aims to investigate the levels and types of PA elements and their association with adverse outcomes in Chinese non-dialysis CKD (ND-CKD) patients.

**Methods and analysis:**

In this prospective cohort study, 374 patients with ND-CKD will be recruited from Guangdong province, South of China. The primary exposure will be levels of PA assessed by ActiGraph GT3X+ accelerometer including the intensity, duration, frequency and type of PA. The traditional Chinese exercises such as tai chi and Baduanjin will also be assessed. The primary outcomes will be all-cause mortality. Other variables including demographics, comorbidities, medication and laboratory markers will be registered. All data will be updated annually for at least 5 years, or until the occurrence of death or initiation of renal replacement therapy. The Spearman correlation coefficient will be used to investigate the correlation between questionnaire-derived and accelerometry-derived PA. The Cox proportional hazards model will be used to investigate the association between level of PA and adverse outcomes. Non-linear associations between PA levels and outcomes, as well as the minimum desirable PA level, will be evaluated using restricted cubic splines.

**Ethics and dissemination:**

The ethical permission for this study was obtained from the ethics committee of Guangdong Provincial Hospital of Chinese Medicine in Guangzhou, China (B2015-152-02). Written informed consent is obtained from all participants. The results will be disseminated by publication in a peer-reviewed journal and presented at relevant conferences.

**STRENGTHS AND LIMITATIONS OF THIS STUDY**

- The study combines both objective measures and validated questionnaires to assess physical activity (PA) elements, significantly enhancing data accuracy.
- The study not only assesses the common physical activities but also a range of traditional Chinese exercises, providing a broader understanding of PA patterns in the population.
- The study investigates various clinically important outcomes, offering a holistic view of the impact of PA on the health of patients with chronic kidney disease.
- The study’s population is primarily from Guangdong province, China, which limits the generalisability of findings to other regions with different climates, lifestyles and exercise habits.

## Introduction

 Chronic kidney disease (CKD) is a significant public health burden worldwide, affecting over 800 million adults or more than 10% of the world’s adult population.^1^ It is associated with a higher risk of all-cause mortality,^2 3^ cardiocerebral vascular disease and hospitalisation.^4^ Physical activity (PA) is a crucial component of lifestyle modification strategies to prevent CKD progression and associated complications.^5 6^ Prior studies have shown that low levels of PA in patients with CKD are linked to adverse outcomes, including poor quality of life, increased risk of cardiovascular disease, hospitalisation and all-cause mortality.^5^

However, most existing studies have relied on subjective questionnaires to assess levels of PA, which are prone to recall bias and social desirability bias,^7^ and most have focused on patients with dialysis and in Western countries.^5 8 9^ The intensity, duration, frequency and types of PA in Chinese patients with non-dialysis CKD (ND-CKD) and their association with adverse outcomes are not well understood. The ActiGraph GT3X+ accelerometer, which is one of the most widely used instruments for assessing levels of PA, makes it possible to record PA levels objectively with high accuracy.^10^

In recent years, traditional Chinese exercise (TCE), a form of mind–body exercise that includes practices such as tai chi, Baduanjin and Wu Qin Xi, has received increasing attention for its potential both mental and physical health benefits.^11^ Studies have demonstrated the positive effects of TCE on various chronic diseases, including cardiovascular disease,^12 13^ hypertension,^14^ type 2 diabetes mellitus,^15^ sarcopenia and frailty.^16^ Nonetheless, research on the impact of TCE on the outcomes of patients with ND-CKD has been limited.

Therefore, this longitudinal cohort study, entitled ‘Physical Activity Elements and Adverse Outcomes in Patients with Chronic Kidney Disease in Guangdong (PEAKING)’ aims to address these knowledge gaps by using both objective and subjective methods to investigate the levels of PA and types of PA such as TCE and their association with outcomes of clinical importance in Chinese patients with ND-CKD. The findings of this study may provide vital information for the development of PA guidelines for patients with CKD in China as well as in other settings that ultimately may improve their prognosis and quality of life.

### Aims

This study aims to achieve the following objectives in Chinese patients with ND-CKD:

1. To comprehensively assess the types, intensity, duration and frequency of PA using both objective and subjective methods.
2. To evaluate the consistency of PA level assessment between subjective measurement by questionnaires and objective measurement by ActiGraph GT3X+ accelerometers.
3. To investigate the impact of levels and types of PA, including TCE, on clinically significant outcomes such as mortality and hospitalisations.
4. To define the minimum level of desirable PA for different types of PA and the cut-off level for PA to be used when evaluating PA level versus mortality.

## Methods and analysis

### Study design and setting

The PEAKING study is a prospective open cohort study and will enrol patients with ND-CKD in the Guangdong Provincial Hospital of Chinese Medicine (GPHCM), which is one of the main referrals and tertiary hospitals of the region, located in Guangzhou, South of China. Guangzhou has a population of approximately 18 million people, making it one of the megacities in China.^17^ This large population provides a diverse pool of potential study participants, ensuring that the study’s findings are representative of the broader CKD population. Additionally, the ageing population, high prevalence of CKD and highly developed economy^18^ in Guangzhou make it an ideal location for the current study.^19 20^ The study is recruiting. The results of this study will be reported according to Strengthening the Reporting of Observational Studies in Epidemiology guidelines.^21^

### Target population and inclusion/exclusion criteria

The study population includes patients with ND-CKD who are attending the nephrology consultation clinic in GPHCM. The study began piloting recruitment of participants since March 2017, allowing us to test the schedule, care processes and patient adherence in real-world practice under our protocol. Recruitment is planned to continue through March 2027. All patients will be followed up for at least 5 years or until the occurrence of death or initiation of renal replacement therapy (RRT).

Patients are eligible for inclusion if they are: (1) older than 18 years; (2) diagnosed with CKD with estimated glomerular filtration rate (eGFR) less than 60 mL/min/1.73 m^2^ or abnormal kidney biomarkers such as proteinuria or haematuria for 90 days or longer according to the Kidney Disease: Improving Global Outcomes guideline.^22^ Exclusion criteria are: (1) patients who had received or are expected to receive RRT including kidney transplantation, haemodialysis and peritoneal dialysis within 1 year; (2) pregnant or lactating women or those planning pregnancy within 1 year; (3) acute myocardial infarction, acute cerebrovascular event or acute obstructive nephropathy, requiring hospitalisation within 3 months prior to recruitment to avoid the impact of these events on the physical fitness and level of PA; (4) severe arrhythmia or heart failure (New York Heart Association class grade III or above), which could not be controlled by medication and at higher risk of deterioration during exercise; (5) active malignant tumour, decompensated cirrhosis or haematopoietic neoplasms, which require specific therapy such as chemotherapy and radiotherapy; (6) serious mental illness or unable to follow the trial protocol; (7) physical disability, such as amputation history.

### Screening and enrolment

**Figure 1:**
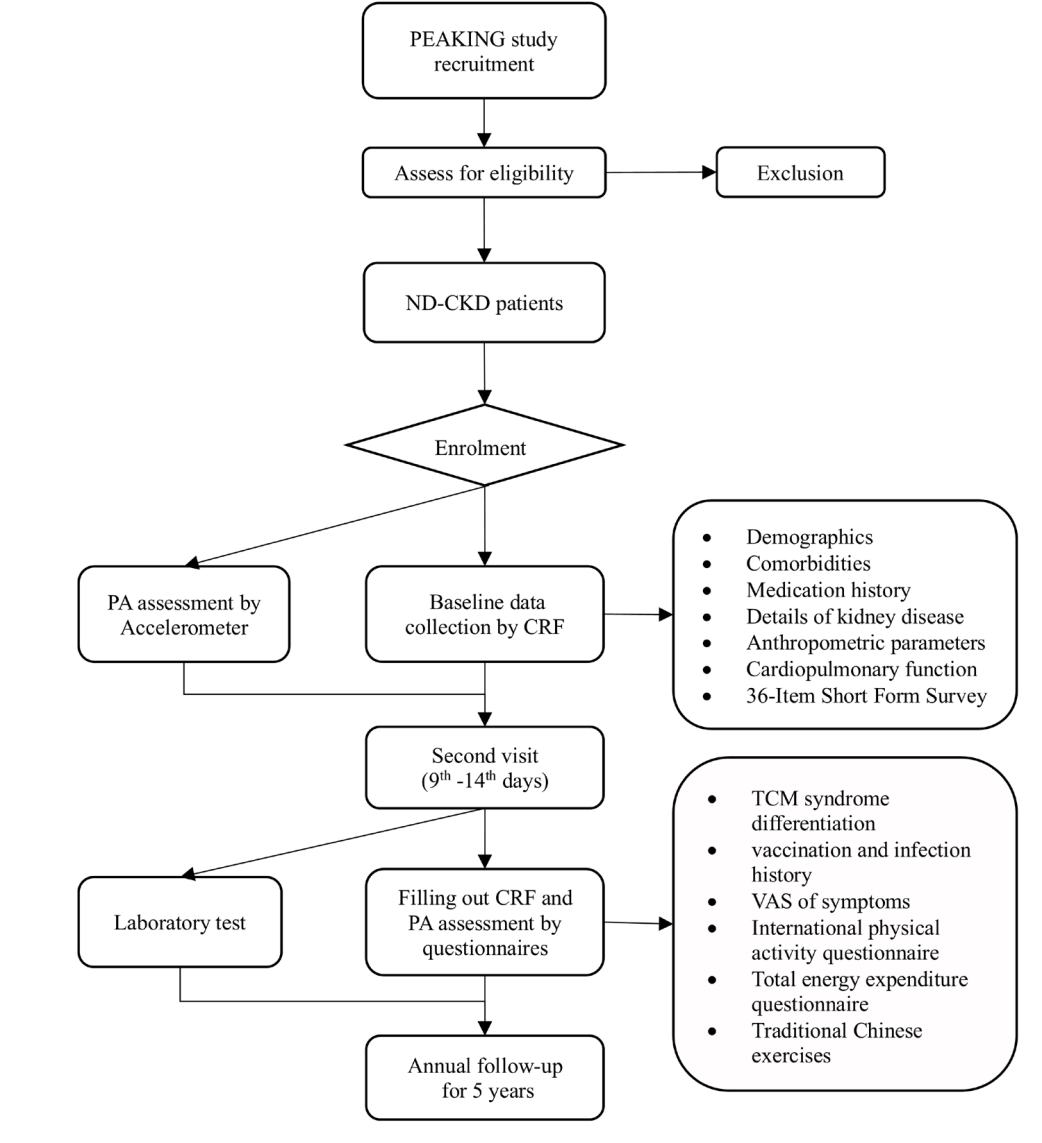
The recruitment process for the PEAKING study. CRF, case report form; ND-CKD, non-dialysis chronic kidney disease; PA, physical activity; PEAKING, Physical Activity Elements and Adverse Outcomes in Patients with Chronic Kidney Disease in Guangdong; TCM, traditional Chinese medicine; VAS, Visual Analog Scale.

Research assistants are responsible for screening and identifying participants from the consultation clinic to ensure their eligibility. After screening, they will explain the study to candidates through a face-to-face interview. Patients who agree to participate in the PEAKING study will sign an informed consent and be assigned a four-digit code as a unique identification for the PEAKING study (figure 1).

### Follow-up and retention

After registration, participants will be required to complete a case report form (CRF) at baseline, providing information on demographics, comorbidities, medication, kidney disease history, anthropometric parameters, cardiopulmonary function and 36-Item Short Form Survey (SF-36). Additionally, they will receive an accelerometer, which will be worn for the next 9 days to record their PA at baseline. Participants will also be scheduled for the second visit.

During the second visit, participants will need to return the accelerometer and complete the remaining part of the CRF, including information on the Traditional Chinese Medicine (TCM) syndrome differentiation (such as the dampness syndrome scale of Chinese medicine) and Visual Analog Scale (VAS) assessment of symptoms and PA function. Additionally, they will undergo PA assessment based on several subjective questionnaires and a series of laboratory tests. Finally, participants will be followed up annually to assess clinical outcomes until the occurrence of kidney failure or death. Details of the follow-up procedure for the PEAKING study are presented in figure 1 and table 1.

**Table 1:** Overview of measurement instruments and time of assessment

Domain	Type	In-person assessment		Distant follow-up				
		Baseline	9th–14th day	1st year	2nd year	3rd year	4th year	5th year
Demographics	Sex, age, ethnicity, marital status, educational level, working status, health insurance, smoking and alcohol drinking habit	√						
Comorbidities	Disease name and course	√			√	√		
Medication history	Drug name, classification, single dose, frequency of administration and route of administration	√			√	√		
Details of kidney disease	Primary disease of CKD, disease duration, and kidney biopsy report, if available	√						
Anthropometric parameters	Body weight, body height, body mass index, waist circumference, handgrip strength and body composition	√			√	√		
TCM syndrome differentiation	Comprehensive analysis of clinical information gained by the four diagnostic TCM procedures: observation, listening, questioning, and pulse analysis by careful self-designed questionnaires		√		√	√	√	√
Vaccination and infection history			√		√	√		
Physical function evaluation	ActiGraph GT3X+	√	√		√	√		
	IPAQ		√		√	√		
	TEEQ		√		√	√		
	TCEs		√		√	√		
Visual Analog Scale	Fatigue, appetite and physical activity		√		√	√		
Cardiopulmonary function	Six-minute walk test	√			√	√		
Laboratory test	Serum tests: complete blood count, sodium, potassium, chloride, calcium, glucose, albumin, creatinine, eGFR, urea nitrogen, uric acid, TC, TG, HDL-C, LDL-C, iPTH;Urinalysis: proteinuria, haematuria and PCR.		√		√	√	√	√
SF-36		√			√	√	√	√
Primary outcomes	All-cause mortality, all-cause hospitalisation			√	√	√	√	√
Secondary outcomes	Infection-related hospitalisation, infection-related mortality, MACEs, MAKEs, quality of life, loss of renal function			√	√	√	√	√

CKD, chronic kidney disease; eGFR, estimated glomerular filtration rate estimated by 2012 CKD-EPI Creatinine Equation; HDL-C, high-density lipoprotein cholesterol; IPAQ, International Physical Activity Questionnaire; iPTH, intact parathyroid hormone; LDL-C, low-density lipoprotein cholesterol; MACEs, major cardiovascular events; MAKEs, major adverse kidney eventPCR, protein-to-creatinine ratio; SF-36, 36-Item Short Form Survey; TC, total cholesterol; TCEs, Traditional Chinese Exercises; TCM, Traditional Chinese Medicine; TEEQ, Total Energy Expenditure Questionnaire; TG, total triglycerides

### Exposure design

#### Primary exposure

##### Level of PA measured by ActiGraph GT3X+

The ActiGraph GT3X+ accelerometer (ActiGraph, LLC Pensacola, Florida, USA) will be used to objectively assess the intensity, duration and frequency of PA. This instrument has been adopted by more than 2000 colleges and institutions as an accurate instrument for evaluation of PA.^23^ It is to be worn on the right hip in the daytime for nine consecutive days but not during bathing or swimming activities.^24^ During this 9-day period, two telephone calls—after the first 24 hours and after the 5th day—will be made to verify the participant’s compliance. The predefined threshold for an acceptable valid wear time shall be set as at least 8 hours per day and with data being collected for a minimum of 3 days (including one non-working day) to be eligible for inclusion in the analysis.^25^ Non-wear time will be defined as a period of at least 60 consecutive minutes for adults aged 18–65 years of zero activity counts and 90 consecutive minutes of zero activity counts for individuals aged 65 years and older, with allowance for up to 2 min of activity counts between 0 and 100.^26^

Data for the ActiGraph will be downloaded and analysed using Actilife software (V.6.12.0). Freedson cut points will be used to convert the raw accelerometer data into intensity bands.^27^ Active activity is categorised into four subcategories: light (100–1951 counts/min), moderate (1952–5124 counts/min), vigorous (5125–9498 counts/min) and very vigorous (>9498 counts/min). Sedentary time shall be quantified using an activity threshold of <100 counts/min. Average time in minutes per day (min/day) will be measured for sedentary time and PA time.^28^ According to the Clinical Practice Guideline of Exercise and Lifestyle in Chronic Kidney Disease, participants will be categorised as active if they meet the current guidelines’ recommendations of 150 min of moderate-intensity PA per week or 75 min of vigorous-intensity activity per week or a combination of moderate and vigorous activity.^29^ Conversely, participants who do not meet these criteria will be categorised as inactive.

### Secondary exposure

#### International Physical Activity Questionnaire (IPAQ)

Participants will report their PA during the last 7 days, including domains of transportation, work, household tasks and leisure time, using the Chinese short-form version of IPAQ (IPAQ-C).^30^ The IPAQ-C records the duration (in minutes) and frequency (days) of walking, as well as moderate-intensity and vigorous-intensity activity. The total metabolic equivalents of task (METs) will be calculated by multiplying the total minutes per week of each activity, resulting in a PA estimation in MET minutes/week. The evaluation for duration and frequency of activities will be taken into account, and the PA will be classified into three levels (low, moderate and high). The validity and reliability of IPAQ-C have been confirmed in patients with ND-CKD based on our previous study using the PEAKING cohort.^31^

#### Total Energy Expenditure Questionnaire (TEEQ)

Participants will complete the Chinese version of the TEEQ (TEEQ-C) to report their daily average energy expenditure and the contribution of PA to their total energy expenditure.^32^ The TEEQ-C considers various types of activities, including sleeping, leisure, household, work and transportation activities. This questionnaire is adapted from the Swedish version of TEEQ. TEEQ-C has been validated and proven reliable for patients with ND-CKD in a previous study using the PEAKING cohort.^32 33^

#### Traditional Chinese Exercises Questionnaire (TCEQ)

The participants will complete a carefully designed TCEQ adapted from IPAQ to assess the type, duration and frequency of common TCE practised in the last 6 months, including Taijiquan (tai chi), Baduanjin (Eight-Section Brocade), Yi Jin Jing (Muscle/Tendon Change Classic), Wu Qin Xi (Five Animals) and Liu Zi Jue (Six Healing Sounds). The validity has been tested in our previous study.^31^

### Outcome measures

#### Primary outcome

The primary outcome will be all-cause mortality, which will be documented using the outcome assessment tool of the CRF during each quarterly visit. If needed, the archives of the medical records from the Hospital Information System and the regional data Centers for Disease Control and Prevention will be referred to verify the outcomes with privacy protection strategy.

#### Secondary outcomes

The secondary outcomes include hospitalisation, cause-specific mortality, major cardiovascular events (MACEs), major adverse kidney events (MAKEs), quality of life and TCM symptom burden. The underlying cause of death or hospital admission was coded by trained nosologists according to the International Classification of Diseases, Tenth Revision (ICD-10). Cardiovascular mortality will be defined as death caused by ischaemic heart disease (I20–I25), heart failure (I11, I13, I50), cerebrovascular disease (I60–I69), arrhythmia (I47–I49) and peripheral artery disease (I70–I79).^34^ Infection-related death will be defined according to the ICD-10, which has been reported in detail in our previous study.^34^ MACEs will be defined as cardiovascular mortality, acute myocardial infarction and stroke, whichever occurred first.^35^ MAKEs will be defined as the initiation of RRT with haemodialysis, peritoneal dialysis and renal transplantation. Quality of life will be measured by SF-36, which is a widely used health survey that covers eight dimensions: physical functioning, social functioning, role limitations due to physical problems, role limitations due to emotional problems, mental health, energy and vitality, pain and general health perceptions.^36^ The symptom burden will be assessed by TCM syndrome differentiation, such as dampness syndrome, and physical functioning assessment.

Other outcomes include the absolute change and slope of eGFR decline during the 5-year follow-up period, absolute changes of urinary protein-to-creatinine ratio, proteinuria, 24-hour urinary protein excretion, intact parathyroid hormone level, serum albumin, etc. Above-mentioned outcome data will be collected through the outcome assessments during each visit by reviewing the laboratory test results and the medical records.

### Physical functioning assessment

Physical functioning assessment will be done when patients return the accelerometer in person. These assessments will be performed annually.

- Handgrip strength will be measured as a proxy for upper limb performance using a digital hand-grip dynamometer (EH101, CAMRY Senssun Weighing Apparatus Group, Guangdong, China), calculated from the highest value of three measurements using participants’ dominant hand. Data will be evaluated and presented for men and women separately.
- Body composition will be measured using bioelectrical impedance analysis (InBody 770; Biospace, Seoul, Korea). This measurement will be performed in the morning, in a fasting state after emptying the bladder. Measurement items of body composition include weight, height, waist-to-hip ratio, fat-free mass, body fat mass, percentage of body fat, visceral fat area, fat mass index, soft lean mass, skeletal muscle mass, total body water, intracellular water and extracellular water. Body mass index will be calculated by dividing weight by the square of height and categorised as severely underweight (<16.5 kg/m^2^), underweight (<18.5 kg/m^2^), normal (18.5–22.9 kg/m^2^) and overweight (>23 kg/m^2^).^37^
- The six-minute walk test, which is a straightforward and non-invasive tool, is to be used to evaluate an individual’s functional capacity to exercise and his/her cardiopulmonary function.^38 39^ During the assessment, the subject is directed to ambulate on a flat and rigid surface for a duration of 6 min, with the aim of achieving maximal distance coverage.
- The blood pressure, heart rate, respiratory rate, Borg Dyspnoea Scale and Borg Rating of Perceived Exertion Scale of the subjects will be recorded both before and after the assessment.
- VAS of symptoms will be used as a subjective rating scale to assess general perceived levels of fatigue, appetite and physical condition in the last week. The scale comprises a straight line ranging from 0 to 10, where score 0 indicates absence of, and score 10 signifies the highest level of the investigated factor, for example, appetite.

### Other covariates or information

A comprehensive classification of demographic characteristics is provided in table 2. The comorbidity profile will be documented and reported using the Charlson Comorbidity Index^40^ and updated annually. Medication data will be renewed annually and categorised according to the Anatomical Therapeutic Chemical classification system developed by the WHO.^41^

**Table 2:** Additional demographic information in the PEAKING study

Demographics	Category
Marital status	Unmarried Married/cohabiting Divorced/single Widowed
Education level	Elementary school Junior high school Senior high school/vocational school Community college Bachelor’s degree Graduate degree or above
Employment	Full-time employed Part-time employed Retired Retired due to illness Laid off Unemployed Student
Occupation (before retirement)	Civil servant Professional/technical staff (teacher, healthcare worker, police, etc) Worker Farmer, forestry worker, fisherman, etc Service industry personnel Student Military personnel Household worker Other
Payment method	Self-support Medical insurance Other (please specify)
Smoking history	Never smoked Quit smoking after previously smoking Currently smoking
Alcohol drinking history	Currently drinking regularly Quit drinking regularly Never drank regularly

PEAKINGPhysical Activity Elements and Adverse Outcomes in Patients with Chronic Kidney Disease in Guangdong

### Sample size estimation

We selected all-cause mortality as the primary study outcome for sample size calculation. The initial sample size estimate of 312 was derived using a free power and sample size calculator (available at http://powerandsamplesize.com). This calculation was based on the following information: the mortality was 11% for patients with CKD with eGFR ranging from 20 to 70 mL/min/1.73 m² who meet the PA guidelines compared with 19% for those who do not, a sample size ratio of 1:1 between exposed and unexposed groups,^42^ a two-sided significance level (α) of 5%, and a study power (1−β) of 80%. To account for potential non-compliance, non-response and loss to follow-up, the sample size was increased by 20%, resulting in a final requirement of 374 patients with CKD.

### Statistical analysis plan

The initial data analysis will be descriptive. Categorical variables will be summarised by frequencies (percentages), while continuous variables will be summarised by both mean and SD for data with normal distributions or median (IQR) for non-normally distributed data. Either a χ^2^ or Fisher’s exact test will be used to compare categorical variables between groups, while Mann-Whitney U test or Student’s t-test will be employed for continuous variables. Repeated measurements of variables obtained at different time points will be analysed using repeated measures Student’s t-test and, if necessary, mixed models.

The Spearman correlation coefficient will be used to compare the consistency between the number of individuals meeting the recommended PA level as determined by the questionnaire and those identified through accelerometer. To interpret the Spearman’s rank correlation coefficient, we will use the following benchmarks: 0–0.20=poor correlation, 0.21–0.40=fair correlation, 0.41–0.60=moderate/acceptable correlation, 0.61–0.80=substantial correlation and 0.81–1.0=near perfect correlation.^43^ Bland-Altman analyses will be used to determine the agreement between questionnaire‐derived and accelerometry‐derived PA. Cox proportional hazard models will be used to investigate the association between levels of PA and adverse outcomes, such as all-cause mortality, with results reported as HR and 95% CI. A Fine and Gray subdistribution hazards model will be employed to account for the competing risk of cause-specific mortality. Poisson regression will be employed to analyse the association between levels of PA and the frequency of adverse outcomes, such as hospitalisation and infection. For longitudinal data, such as the absolute change and slope of eGFR, a linear mixed effects model will be applied. Additionally, non-linear associations between PA levels and outcomes, as well as the minimum desirable PA level, will be evaluated using restricted cubic splines. Covariates for the models will be selected based on prior knowledge and published papers. Patients will be followed from the physical assessment date at baseline until the occurrence of death or end of follow-up, whichever occurred first.

Even with some retention strategies, considerable missing data is expected over 5-year follow-up. The proportion of missing data will be summarised in each group and at each visit point. If less than 20% of the data is missing for the covariate data, we will perform a complete case analysis. If there is more than 20% of missing data, we will perform Little’s test and use multiple imputations under the assumption that missing is at random. Subgroup analyses will be conducted across baseline eGFR levels, demographics and other factors, if possible. A two-sided p value<0.05 will be considered statistically significant. All statistical analyses will be performed using R (V.4.1.1; R Foundation for Statistical Computing, Vienna, Austria).

### Patient and public involvement

The design of the current study did not involve participants directly. However, the research coordinators will maintain contact with participants through email, telephone or social media, and we will collect feedback during the study procedure. The study was initially designed by the research group comprising clinicians, nurses and researchers who work clinically with individuals with CKD. During the recruitment phase, a collaboration was established with representatives from interest organisations in China. This collaboration is ongoing with regular meetings.

## Ethics and dissemination

The study is based on informed written consent, and participants can withdraw from the study at any point in time. Ethical permission for this study was obtained from the ethics committee of GPHCM in Guangzhou, China (B2015-152-02). The results of the study will be presented at national and international conferences and published in peer-reviewed journals.
