# Supplementary material for: Physical Activity Elements and Adverse Outcomes in Patients with Chronic Kidney Disease in Guangdong (PEAKING) project: protocol for a prospective cohort study
Source: BMJ Open. 2024 Oct 22;14(10):e086509. doi: 10.1136/bmjopen-2024-086509 (PMC11499868; doi:10.1136/bmjopen-2024-086509)
Supplement: Supplementary file 1 [file bmjopen-2024-086509.reviewer_comments.pdf]

PEER REVIEW HISTORY

BMJ Open publishes all reviews undertaken for accepted manuscripts. Reviewers are asked to complete a checklist review form and are provided with free text boxes to elaborate on their assessment. These free text comments are reproduced below.

ARTICLE DETAILS

Title (Provisional)

Physical Activity Elements and Adverse Outcomes in Patients with Chronic Kidney Disease in Guangdong (PEAKING) Project: protocol for a prospective cohort study

Authors

Yang, Changyuan; Duan, Ruolan; Yang, Zhenhua; Qiu, Jiamei; Pi, Minhui; Ling, Xitao; Xiao, Cuixia; Zeng, Jiahao; He, Jiawei; Huang, Jiasheng; Zhang, La; Qin, Xindong; Tang, Fang; Fu, Li-zhe; Hou, haijing; Liu, Xusheng; Lindholm, Bengt; Lu, Fuhua; Wu, Yifan; Su, Guobin

---

VERSION 1 - REVIEW

**Reviewer**	**1**
**Name**	**Rahmel, Tim**
**Affiliation**	**Universitätsklinikum Knappschaftskrankenhaus Bochum**
**Date**	**15-May-2024**
**COI**	**None**

This is an very interesting study protocol regarding an potentially clinical important improvement how to assess the physical activity (PA) of patients with CKD. Here the authors introduce a potentially superior method to assess PA by using the "Actigraph GT3X+ accelerometer".

However, my subsequent points should be addressed to improve clarity of the reviewed study protocol.

Page 8 Line 40 “ March 2017 to March 2027” (sic!) How can the recruitment period of a prospective study start in the past? Please clarify context of the mentioned period or correct the recruitment dates.

The selection of the reference group seems not directly linked to the primary study objective. Why should PA not or be less relevant in group of patients with CKD 1-2? Why should it not be possible to analyse CKD 1-2 patients with the "Actigraph GT3X+ accelerometer" tool? Thus, it is not clear to me, which study questions will be answered with this reference group? As you state "serve as a reference group for comparisons with our cohort" - you should clarify for your readers the specific questions and hypotheses that explain and justifies the specific reference group more clearly.

The number of cases seems relatively low to assess the primary objective. This is defined as the ability of the "Actigraph GT3X+ accelerometer" to adequately assess PA and being superior compared to conventional questionnaires in terms of diagnostic accuracy. However, the case number planning is solely focussed on the endpoint of the difference in mortality between patients with good PA and bad PA. However, the required sample sizes for the study should also consider different levels of accuracies and marginal purpose of testing tool as well. Hence, the sample size should vary with the accuracy index and likewise the estimated effect size of interest. Thus, I expect an overestimation of the effect size in the current version and consequently the sample size may not being sufficient to assess the primary objective.

In consideration of the aforementioned limitations. The primary/secondary study objectives and the respective outcome measure seems not perfectly aligned.

If I get the objective right the authors want to test the hypothesis that the assessment of PA is superior (in terms of accuracy regarding mortality prediction), but this is not clearly reflected in the subsequent sample size calculation (as mentioned above) and also in the statistical analysis plan. Here the authors solely focus on the impact of PA on mortality. However, the latter question is already clary answered by previous studies (as also stated by the authors in their introduction).

**Reviewer**	**2**
**Name**	**Pou, Sonia Alejandra**
**Affiliation**	**CONICET Cordoba, Research Institute of Health Sciences**
**Date**	**22-Aug-2024**
**COI**	**None.**

This study aims to examine the levels and types of physical activity (PA) (including traditional exercise practices in China) among patients with ND-CKD in Guangdong province, southern China, assessing their association with clinically important outcomes such as mortality and hospitalizations, among others. The proposal is interesting as it addresses a knowledge gap

in the field of nephrology, offering potential value for public health by generating useful information for developing physical activity guidelines tailored to CKD patients in the Chinese context. A notable strength of this work is its longitudinal study design (a gold standard in epidemiology for assessing health risks), and the comprehensive approach the authors take, incorporating diverse methodologies and addressing multiple factors.

However, several points need to be clarified to enhance understanding of the study's rationale and methodology. Accordingly, I have some major and minor comments:

Major comments:

-Title: The terms 'Physical Evaluation' and 'Physical Activity' do not appear to be equivalent. This observation is based on the 'Physical Functioning Assessment' section of the manuscript, where the authors detail that the proposed physical evaluation is distinct from the assessment of physical activity as such. Consequently, I suggest reviewing the consistency between these terms in the title and the proposed objectives. In my opinion, considering the objectives as they are presented (with a focus on PA as the main outcome), the title should refer to PA. The physical evaluation can still be included in the methods section as proposed (as a secondary outcome).

- In the Abstract, there is a lack of information regarding the statistical analysis plan. Additionally, here and in the manuscript text, I suggest reviewing whether the initiation of renal replacement therapy is actually a primary outcome.

- Quality of life was highlighted as an outcome in the Strengths and limitations of this study section. However, the Methods section does not provide details regarding this variable and its evaluation procedure. It would be desirable to include more methodological details on this matter. It is only mentioned that "quality of life was measured by SF-36."

-The second paragraph of the Statistical Analysis Plan section needs to be reviewed. I suggest organizing the analysis process by more clearly outlining the rationale or objectives of the proposed analyses. The range of analyses is presented somewhat vaguely or disconnectedly at times. Consider for example that the Cox regression model allows for the calculation of hazard ratios; linear mixed effects models will be applied to the longitudinal data, with logistic or Poisson regression being cases within this framework, etc.

Minor comments:

-The acronym ESKD should be spelled out upon its first mention.

-The authors state: 'The major secondary outcomes include all-cause and cause-specific hospitalization and mortality.' However, since all-cause mortality was referred to as a primary outcome, there may be an error here. Additionally, in the literature on causes of mortality, it is common practice to report ICD-10 codes to clarify classification criteria. Therefore, I suggest incorporating these codes if possible.

-In the 'Physical Functioning Assessment' section, the procedure and instruments for measuring Body Mass Index (BMI) should be detailed, as with the other variables, especially if it will be measured anthropometrically.

- Where it says 'Cox proportional hazards model or a competitive risk model,' did you mean 'proportional risk model'? Competitive risk models do not seem to address the proposed objective. If you are referring to them, please clarify their rationale.

VERSION 1 - AUTHOR RESPONSE

Reviewer: 1

Dr. Tim Rahmel, Universitätsklinikum Knappschaftskrankenhaus Bochum

Comments to the Author:

This is an very interesting study protocol regarding an potentially clinical important improvement how to assess the physical activity (PA) of patients with CKD. Here the authors introduce a potentially superior method to assess PA by using the "Actigraph GT3X+ accelerometer". However, my subsequent points should be addressed to improve clarity of the reviewed study protocol.

Dear Dr. Tim Rahmel,

Thank you for your encouraging feedback and valuable suggestions regarding our manuscript. We appreciate your recognition of the clinical significance of our study.

Please find the point-by-point responses below.

Note: All index numbers in this file are based on the latest version of the manuscript (with track changes).

Page 8 Line 40 " March 2017 to March 2027" (sic!) How can the recruitment period of a prospective study start in the past? Please clarify context of the mentioned period or correct the recruitment dates.

Response: Thank you for bringing this point up. We understand the confusion regarding the recruitment period for a prospective study, and we have revised the relevant description. Actually, this study is indeed prospective. In order to prepare for a longer and consistence cohort, we have

piloted to recruit patients from March 2017 and planned to continue through March 2027 or even longer. This allowed us to test the schedule, process of care and patient's adherence in real-world practice under our protocol. At the same time, this approach enables the study to ensure a sufficient sample size over a relatively long period.

"The study population includes patients with ND-CKD who are attending the nephrology consultation clinic in GPHCM. The study began piloting recruitment of participants since March 2017, allowing us to test the schedule, care processes, and patient adherence in real-world practice under our protocol. *Recruitment is planned to continue through March 2027."* (Page 6 line 30).

The selection of the reference group seems not directly linked to the primary study objective. Why should PA not or be less relevant in group of patients with CKD 1-2? Why should it not be possible to analyse CKD 1-2 patients with the "Actigraph GT3X+ accelerometer" tool? Thus, it is not clear to me, which study questions will be answered with this reference group? As you state "serve as a reference group for comparisons with our cohort" - you should clarify for your readers the specific questions and hypotheses that explain and justifies the specific reference group more clearly.

Response: We apologize for the confusion. Actually, both patients with CKD stage 3-5 and 1-2 will be assessed by the Actigraph GT3X+ accelerometer in the current study. The term "reference group" does not refer to a control group; instead, it's intended to represent a pool within our target population. The reason why we divided the population into two groups was the hypothesis that patients with advanced CKD were more likely to develop adverse outcomes compared with those with earlier stage CKD.

To avoid confusion, we have removed the term "reference population" and the description of this group. Patients with both earlier and advanced CKD will be considered as our target population. We will conduct subgroup analyses based on CKD stage to address this difference (Page 7 reference population section).

~~*Reference population*~~

~~*The target population in our study was patients with ND-CKD stage 3-5 for their higher progression risk of ESKD. In order to compare the level and types of PA in patients with different stages of ND-CKD, we also include a subset of population with CKD stage 1-2 as the reference population in our initial screening and invite them to participate. Once the eGFR decline below 60 ml/min/1.73m2, we will follow the schedule of the PEAKING cohort for these patients. Thus, this reference population can be considered as a pool of potential candidates that may be included as a formal PEAKING participant and also can serve as a reference group for comparisons with our cohort.*~~

The number of cases seems relatively low to assess the primary objective. This is defined as the ability of the "Actigraph GT3X+ accelerometer" to adequately assess PA and being superior compared to conventional questionnaires in terms of diagnostic accuracy. However, the case number planning is solely focussed on the endpoint of the difference in mortality between patients with good PA and bad PA. However, the required sample sizes for the study should also consider different levels of accuracies and marginal purpose of testing tool as well. Hence, the sample size should vary with the accuracy index and likewise the estimated effect size of interest. Thus, I expect an overestimation of the effect size in the current version and consequently the sample size may not being sufficient to assess the primary objective.

Sample size estimation (Page 11):

Response: We recalculated the sample size considering both objectives, as per your suggestions. For the first aim, we selected all-cause mortality as the primary study outcome for sample size calculation, resulting in an initial estimate of 312 participants. For the second aim, which involves evaluating the consistency of PA level assessment between subjective measurement by IPAQ and objective measurement by ActiGraph GT3X+ accelerometers, we considered a correlation coefficient (r) of 0.4 for moderate-to-vigorous physical activity between IPAQ and accelerometers, based on previous studies where it ranged from 0.4 to 0.6[1 2]. A two-sided significance level (α) of 5% and a study power (1-β) of 80% were used, resulting in a sample size estimate of 46 participants. To account for potential non-compliance, non-response, and loss to follow-up, the sample size was increased by 20%. In summary, we plan to recruit 374 CKD patients to ensure the sample size meets all the study objectives.

"We selected all-cause mortality as the primary study outcome for sample size calculation. The initial sample size estimate of 312 was derived using a free power and sample size calculator (available at http://powerandsamplesize.com). This calculation was based on the following information: the mortality was 11% for CKD patients with eGFR ranging from 20–70 ml/min/1.73m² who meet the PA guidelines compared to 19% for those who do not, a sample size ratio of 1:1 between exposed and unexposed groups[3], a two-sided significance level (α) of 5%, and a study power (1-β) of 80%. To account for potential non-compliance, non-response, and loss to follow-up, the sample size was increased by 20%, resulting in a final requirement of 374 CKD patients."

In consideration of the aforementioned limitations. The primary/secondary study objectives and the respective outcome measure seems not perfectly aligned.

Response: We have revised the aims to ensure that they align with methods and outcomes section.

1. *To comprehensively assess the types, intensity, duration, and frequency of PA using both objective and subjective methods.*
2. *To evaluate the consistency of PA level assessment between subjective measurement by questionnaires and objective measurement by ActiGraph GT3X+ accelerometers.*
3. *To investigate the impact of levels and types of PA, including TCE, on clinically significant outcomes such as mortality and hospitalizations.*
4. *To define the minimum level of desirable PA for different types of PA and the cut-off level for PA to be used when evaluating PA level vs. mortality.*

If I get the objective right the authors want to test the hypothesis that the assessment of PA is superior (in terms of accuracy regarding mortality prediction), but this is not clearly reflected in the subsequent sample size calculation (as mentioned above) and also in the statistical analysis plan. Here the authors solely focus on the impact of PA on mortality. However, the latter question is already clary answered by previous studies (as also stated by the authors in their introduction).

Response: Yes, we agree with you. We have rewritten the aims and statistical analysis plan section, and the sample size has been recalculated as suggested. Our second goal is to evaluate the consistency of PA level assessment between subjective measurement by questionnaires and objective measurement by ActiGraph GT3X+ accelerometers. And then we will investigate the impact of different levels and types of PA, as measured by the accelerometer (the golden standard method), on a range of clinically significant outcomes.

An additional advantage of this study is that we will also assess the impact of several types of traditional Chinese exercises, such as Taichi, on aforementioned outcomes. In addition, we aim to define the minimum level of desirable PA for different types of PA and the cut-off level for PA (based on accelerometer data) to be used when evaluating PA level vs. mortality, as there is limited evidence available on this.

Aims (Page 4):

1. *To comprehensively assess the types, intensity, duration, and frequency of PA using both objective and subjective methods.*
2. *To evaluate the consistency of PA level assessment between subjective measurement by questionnaires and objective measurement by ActiGraph GT3X+ accelerometers.*
3. *To investigate the impact of levels and types of PA, including TCE, on clinically significant outcomes such as mortality and hospitalizations.*
4. *To define the minimum level of desirable PA for different types of PA and the cut-off level for PA to be used when evaluating PA level vs mortality.*

Statistical analysis plan (Page 12):

"The Spearman correlation coefficient will be used to compare the consistency between the number of individuals meeting the recommended PA level as determined by the questionnaire and those identified through accelerometer. To interpret the Spearman's rank correlation coefficient, we will use the following benchmarks: 0-0.20 = poor correlation, 0.21–0.40 = fair correlation, 0.41–0.60 = moderate/acceptable correlation, 0.61–0.80 = substantial correlation, and 0.81–1.0 = near perfect correlation[4]*. Bland–Altman analyses will be used to determine the agreement between questionnaires - and accelerometry -derived PA."*

Reviewer: 2

Prof. Sonia Alejandra Pou, CONICET Cordoba, National University of Cordoba

Comments to the Author:

This study aims to examine the levels and types of physical activity (PA) (including traditional exercise practices in China) among patients with ND-CKD in Guangdong province, southern China, assessing their association with clinically important outcomes such as mortality and hospitalizations, among others. The proposal is interesting as it addresses a knowledge gap in the field of nephrology, offering potential value for public health by generating useful information for developing physical activity guidelines tailored to CKD patients in the Chinese context. A notable strength of this work is its longitudinal study design (a gold standard in epidemiology for assessing health risks), and the comprehensive approach the authors take, incorporating diverse methodologies and addressing multiple factors.

However, several points need to be clarified to enhance understanding of the study's rationale and methodology. Accordingly, I have some major and minor comments:

Dear Prof. Sonia Alejandra Pou,

Thank you very much for your encouraging feedback and recognition regarding our manuscript. We really appreciate it. Please find the point-by-point responses below.

Note: All index numbers in this file are based on the latest version of the manuscript (with track changes).

Major comments:

-Title: The terms 'Physical Evaluation' and 'Physical Activity' do not appear to be equivalent. This observation is based on the 'Physical Functioning Assessment' section of the manuscript, where the authors detail that the proposed physical evaluation is distinct from the assessment of physical activity as such. Consequently, I suggest reviewing the consistency between these terms in the title and the proposed objectives. In my opinion, considering the objectives as they are presented (with a focus on PA as the main outcome), the title should refer to PA. The physical evaluation can still be included in the methods section as proposed (as a secondary outcome).

Response: We have revised the title to clearly reflect "Physical Activity" as the primary exposure, as suggested.

"Physical Activity Elements and Adverse Outcomes in Patients with Chronic Kidney Disease in Guangdong (PEAKING) Project: Protocol for A Prospective Cohort Study"

- In the Abstract, there is a lack of information regarding the statistical analysis plan. Additionally, here and in the manuscript text, I suggest reviewing whether the initiation of renal replacement therapy is actually a primary outcome.

Response: we have incorporated the statistical analysis plan into the abstract section, as suggested (Page 3).

"The Spearman correlation coefficient will be used to investigate the correlation between questionnaires and accelerometry-derived PA. The Cox proportional hazards model will be used to investigate the association between level of PA and adverse outcomes. The non-linear associations and the minimum desirable level of PA with outcomes will be assessed using restricted cubic splines."

- Quality of life was highlighted as an outcome in the Strengths and limitations of this study section. However, the Methods section does not provide details regarding this variable and its evaluation procedure. It would be desirable to include more methodological details on this matter. It is only mentioned that "quality of life was measured by SF-36."

Response: we agree with your suggestions and added more detailed description of quality of life (Page 10 Line 17).

"Quality of life will be measured by SF-36, which is a widely utilized health survey that covers eight dimensions: physical functioning, social functioning, role limitations due to physical problems, role limitations due to emotional problems, mental health, energy and vitality, pain, and general health perceptions"

-The second paragraph of the Statistical Analysis Plan section needs to be reviewed. I suggest organizing the analysis process by more clearly outlining the rationale or objectives of the proposed analyses. The range of analyses is presented somewhat vaguely or disconnectedly at times. Consider for example that the Cox regression model allows for the calculation of hazard ratios; linear mixed

effects models will be applied to the longitudinal data, with logistic or Poisson regression being cases within this framework, etc.

Response: we have rewritten the Statistical Analysis Plan section to more clearly outline the rationale and objectives of each proposed analysis, as suggested (Page 12).

"Cox proportional hazards models will be used to investigate the association between levels of PA and adverse outcomes, such as all-cause mortality, with results reported as hazard ratio (HR) and 95% confidence intervals (95%CI). A Fine and Gray subdistribution hazards model will be employed to account for the competing risk of cause-specific mortality. To analyze the association between levels of PA and the frequency of adverse outcomes, such as hospitalization and infection, Poisson regression will be employed. For longitudinal data, such as the absolute change and slope of eGFR, a linear mixed-effects model will be applied. Additionally, non-linear associations between PA levels and outcomes, as well as the minimum desirable PA level, will be evaluated using restricted cubic splines (RCS). Covariates for the models will be selected based on prior knowledge and published papers."

Minor comments:

-The acronym ESKD should be spelled out upon its first mention.

Response: we have replaced the term "ESKD" with "kidney failure" throughout the entire manuscript. According to the latest KDIGO guideline, the term ESKD or ESRD should no longer be used.

Kidney Disease: Improving Global Outcomes (KDIGO) CKD Work Group. KDIGO 2024 Clinical Practice Guideline for the Evaluation and Management of Chronic Kidney Disease. Kidney Int. 2024 Apr;105(4S):S117-S314.

-The authors state: 'The major secondary outcomes include all-cause and cause-specific hospitalization and mortality.' However, since all-cause mortality was referred to as a primary outcome, there may be an error here. Additionally, in the literature on causes of mortality, it is common practice to report ICD-10 codes to clarify classification criteria. Therefore, I suggest incorporating these codes if possible.

Response: we have clarified the classification criteria for cause-specific mortality, as suggested (Page 9-10 line 38).

"The underlying cause of death or hospital admission was coded by trained nosologists according to the International Classification of Diseases (ICD), Tenth Revision. Cardiovascular mortality will be defined as death caused by ischaemic heart disease (I20–I25), heart failure (I11, I13, I50), cerebrovascular disease (I60–I69), arrhythmia (I47-I49), and peripheral artery disease (I70–I79)[5]. Infection-related death will be defined according to the ICD-10, which has been reported in detail in our previous study[5]. MACEs will be defined as cardiovascular mortality, acute myocardial infarction, and stroke, whichever occurred first.[6] "

-In the 'Physical Functioning Assessment' section, the procedure and instruments for measuring Body Mass Index (BMI) should be detailed, as with the other variables, especially if it will be measured anthropometrically.

Response: In this study, body composition will also be measured using bioelectrical impedance analysis (InBody 770; Biospace Co., Ltd, Seoul, Korea). Therefore, we moved BMI description to follow the "Body composition measurement" section to ensure clear (Page 11 line 12).

"Body composition will be measured using bioelectrical impedance analysis (InBody 770; Biospace Co., Ltd, Seoul, Korea). This measurement will be performed in the morning, in a fasting state after emptying the bladder. Measurement items of body composition include weight, height, waist-to-hip ratio, fat free mass, body fat mass, percentage of body fat, visceral fat area, fat mass index, soft lean mass, skeletal muscle mass, total body water, intracellular water and extracellular water. Body mass index (BMI) will be calculated by dividing weight by the square of height (kg/m^2) and categorized as severely underweight (<16.5kg/m2), underweight (<18.5kg/m2), normal (18.5-22.9 kg/m2), and overweight (>23 kg/m2)[35]."

- Where it says 'Cox proportional hazards model or a competitive risk model,' did you mean 'proportional risk model'? Competitive risk models do not seem to address the proposed objective. If you are referring to them, please clarify their rationale.

Response: We plan to use Cox proportional hazards model to investigate the association between levels of PA and risk of adverse outcomes, such as all-cause mortality. Risk of cause-specific death will be examined by a competing risk analysis (Fine and Gray subdistribution hazards models). We have rewritten this section to ensure clarity (Page 13 line 1).

"Cox proportional hazards models will be used to investigate the association between levels of PA and adverse outcomes, such as all-cause mortality, with results reported as hazard ratio (HR) and 95% confidence intervals (95%CI). A Fine and Gray subdistribution hazards model will be employed to account for the competing risk of cause-specific mortality."

*** ***

COI statements:

Reviewer: 1

Competing interests of Reviewer: None.

Reviewer: 2

Competing interests of Reviewer: None.

References:

1. Macfarlane DJ, Lee CCY, Ho EYK, et al. Reliability and validity of the Chinese version of IPAQ (short, last 7 days). *Journal of Science and Medicine in Sport* 2007;10(1):45-51. doi: 10.1016/j.jsams.2006.05.003
2. Cleland C, Ferguson S, Ellis G, et al. Validity of the International Physical Activity Questionnaire (IPAQ) for assessing moderate-to-vigorous physical activity and

sedentary behaviour of older adults in the United Kingdom. *BMC Med Res Methodol* 2018;18(1):176. doi: 10.1186/s12874-018-0642-3 [published Online First: 20181222]
3. Ricardo AC, Anderson CA, Yang W, et al. Healthy Lifestyle and Risk of Kidney Disease Progression, Atherosclerotic Events, and Death in CKD: Findings From the Chronic Renal Insufficiency Cohort (CRIC) Study. *American journal of kidney diseases : the official journal of the National Kidney Foundation* 2015;65(3):412-24. doi: 10.1053/j.ajkd.2014.09.016
4. Sedgwick P. Spearman's rank correlation coefficient. *Bmj* 2014;349:g7327. doi: 10.1136/bmj.g7327 [published Online First: 20141128]
5. Su G, Xu Y, Xu X, et al. Association between reduced renal function and cardiovascular mortality in patients hospitalized with infection: A multi-center cohort study. *Eur J Intern Med* 2018;57:32-38. doi: 10.1016/j.ejim.2018.06.017 [published Online First: 20180628]
6. Bosco E, Hsueh L, McConeghy KW, et al. Major adverse cardiovascular event definitions used in observational analysis of administrative databases: a systematic review. *BMC Med Res Methodol* 2021;21(1):241. doi: 10.1186/s12874-021-01440-5 [published Online First: 20211106]

VERSION 2 - REVIEW

**Reviewer**	**1**
**Name**	**Rahmel, Tim**
**Affiliation**	**Universitätsklinikum Knappschaftskrankenhaus Bochum**
**Date**	**06-Oct-2024**
**COI**	

All issues raised were addressed sufficiently.

**Reviewer**	**2**
**Name**	**Pou, Sonia Alejandra**
**Affiliation**	**CONICET Cordoba, Research Institute of Health Sciences**
**Date**	**18-Sep-2024**
**COI**	

The authors have successfully addressed the previous comments, and I consider this work suitable for publication.

I suggest a minor correction in the Abstract: replace 'AND' with 'OF' in the following sentence: 'The non-linear associations of the minimum desirable level of PA with outcomes will be assessed using restricted cubic splines.'
